# Good clinical practice in clinical interventional studies

**DOI:** 10.3402/ecrj.v1.26422

**Published:** 2014-12-12

**Authors:** Herman Pieterse, Zuzana Diamant

**Affiliations:** 1 Department of Pharmacology, Heymans Institute, University of Ghent, Ghent, Belgium; 2 Department of Respiratory Medicine & Allergology, Skane University, Lund, Sweden; 3 Department of General Practice and QPS-NL, University Medical Centre Groningen, Groningen, The Netherlands

**Keywords:** clinical interventional studies, quality, safety, GCP, clinical trials

## Abstract

Good clinical practice (GCP) guidelines should always be implemented and obeyed in clinical interventional studies. In this mini-review, we will address several burning questions relating to GCP in a concise ‘frequently asked questions’ format. While compliance to current rules and regulations is our mission, we also wish to play devil's advocate attempting to translate the rules into sizeable chunks using a high dose of common sense.

Good clinical practice (GCP) guidelines (1) should always be implemented and obeyed in clinical interventional studies (2, 3). In this mini-review, we will address several burning questions relating to GCP in a concise ‘frequently asked questions’ (FAQ) format. While compliance to current rules and regulations is our mission, we also wish to play devil's advocate attempting to translate the rules into sizeable chunks using a high dose of common sense.

## How to conduct a clinical interventional study in compliance with GCP?

Everyone participating in a clinical study bears the responsibility to conduct the study in compliance with the rules and regulations. Both the investigator, his or her team, the management of the hospital, and the sponsor should obey the basic rules of safety, accuracy, and reliability in all fairness according to common sense. These basic rules comprise:

1. Always document whatever is agreed with whom, for when.
2. Any agreement can be changed in time, but it is evident that this change should be documented in order to avoid any misunderstanding.

The design, review, and approval of standard essential documents (e.g. how to design a proper patient information folder?) should be described in a standard operational procedure (SOP). When sponsors of a clinical study do not work according to predefined standard operational procedures embedded in a quality system, then future legal action cannot be avoided. This also applies to clinical research with medical devices, food supplements and other nutraceuticals, or psychological experiments. Common sense dictates that any research should be done in compliance with the best practices for both scientific, ethical, and moral standards. A risk-based quality control (i.e. monitoring) should be executed to prove that credible data have been collected reliably.

1. GCP is a quality system, consisting of a set of standards ensuring adequate protection for individuals participating in clinical studies concerning their privacy, safety, rights, and wellbeing. Moreover, by implementing GCP into an organization, a statement is made that the conduct of the study is such that adequate and appropriate data will be collected in a reliable manner.
2. Fore-warned is fore-armed, that is, everyone involved in clinical research or in patient care should draft a quality system to describe the key tasks that should be conducted in the research process, including a delegation log as to who within the organization is doing what, when, and how?

## Phases of the clinical research process

The clinical research process can be subdivided into three phases:

1. The preparation phase: In this phase, all procedures necessary to prepare the study will be well described, that is, from study outline to study protocol, from patient information leaflet to informed consent form, from risk analysis to investigator's drug or device brochure, and from summary of product characteristics to investigational medicinal (or device) product dossier. Furthermore, how to apply for an ethics committee approval, resource management and workload calculations, and the drafting of a project plan and financing will be done in this phase.
2. The execution phase: This phase includes delegation and assignment to study specific tasks, as to who will do what, how, with whom, by when, and so on. Moreover, in this phase, a monitor manual including the extent of in-process quality control (monitoring) will be defined and how to report which adverse event to whom by when. It is recommended to describe any unanticipated situation in a project manual.
3. Data analysis and reporting phase: How to analyze the data will be described in data management manual including instructions on how to collect and database the data and how to clean the data. For that purpose, a data validation plan will be drafted. The statistical analysis will be described in a statistical analysis plan (SAP) that has been approved prior to the execution of the SAP. The study report not only describes the results but also the process with an accurate account of all patients involved in the study (safety population, intention to treat population, and the per protocol population).

A number of supporting processes should also be described. In a quality manual, the business process will be described, including the quality system. Also, the quality manual would address aspects such as how to conduct quality assurance activities, namely auditing and process improvement processes, and how to survive a regulatory inspection. A definite part of the supporting documentation is the SOP on how to train, educate, and qualify personnel. If tasks will be delegated to third parties, then a SOP on how to select, qualify, and guide a third party is a prerequisite. And last but not least, clinical research involves working with people, and it is wise to prepare for the worst scenario. This means that a SOP on how to deal with suspicion for fraud or misconduct should be detailed. Table 1 shows FAQ on various aspects of clinical interventional trials.

1. The design and maintenance of a simple but effective quality system is an absolute condition to conduct the study in compliance with the regulatory requirements to ensure the public that the safety of participating subjects will be looked after, and to ensure that everyone has performed his or her tasks in the most accurate, honest, and fair way.

**Table 1:** Frequently asked questions, answers, and tips for interventional clinical trials referring to GCP^a^

Question	Answer	ICH GCP reference
1. When is an investigator qualified to conduct a study?	Legislation and regulations require that all parties involved in clinical research are qualified by education and experience and only perform their study-related roles and tasks after thorough theoretical and/or practical training. The investigator and his/her team, the sponsor's employees, and the members of independent ethics committees should have the knowledge and experience to fulfill their tasks adequately as required by local and international regulations and guidelines, as well as their organization's SOPs. • Qualification means having the right people in the right place at the right time to jointly ensure that a clinical study is conducted properly and efficiently in an atmosphere of cooperation and with a lot of common sense.	4.1.1.
2. Under which conditions can the investigator delegate tasks to his investigational team?	A clinical trial is a complex enterprise. The primarily responsible principal investigator will delegate a lot of tasks to designees. However, delegation does not mean ‘shifting’ the task. Although tasks can be delegated to qualified and competent employees, the principal investigator should keep a proper oversight of the study. • At least one to two times, the principal investigator should verify how the employee conducts the delegated task and should document the findings (e.g. by signing the documents).	4.2.4.
3. How to ensure that key members of the investigational team in a clinical study are qualified and knowledgeable?	The knowledge and experience of all parties involved must be fully documented at all times. The actual principal investigator and sponsor are obliged to maintain a track record of the knowledge and experience of the persons who have at one point or another been involved in the conduct of a clinical study. • A complete systematic archive (updated and signed CV, training record) should therefore be maintained of all employees involved in clinical research including their educational background, their work experience, and other relevant information, such as attendance of specific courses, conferences, and workshops (including all necessary certificates). In addition to a thorough basic education, each employee – both of the sponsor and of the investigational team – including the investigator – should be familiar with all applicable regulations and guidelines concerning the conduct of clinical research. This means that everybody should have working knowledge of the ICH guideline for GCP, the FDA CFR, the European Directive for Clinical Research, as well as of local regulations. • A refresher course on GCP, rules and regulations should be followed at least once every 4 years unless pivotal rules have been modified in the meantime.	4.2.3., 8.3.24
4. What does ‘accurate working in clinical research’ implicate for the investigator?	Various national and international laws, directives, rules, regulations, and guidelines specify requirements concerning the carefulness or accuracy according to which clinical research should be conducted. In this context, it is, however, not easy to define the term ‘acting carefully’ in a quantitative manner. Accuracy means with care, keeping a watchful eye on any disorder or discrepancy, acting properly and cautiously. • Acting carefully is a matter of mind set. Especially when designing and preparing a clinical study, all parties seem to be beating about the bush rather than acting in a straightforward fashion. Both the sponsor and the investigator seem more interested in seeing the things they like to see and hearing the things they like to hear – selective perception – than in accepting the full consequences of everything that a clinical study involves. • Failure of either party to accept important issues will always result in serious problems during the conduct of the study, either at the start or at a later stage.	4.1.
	When the monitor, appointed by the sponsor, very much wants to select a certain physician as an investigator for a study, (s)he often does not properly interpret the – verbal and non-verbal – signals that the investigator is transmitting concerning the study and his/her willingness to participate. The monitor only hears what (s)he wants to hear, because it is important for one reason or another to have this particular physician in the study. And, the master's eye makes the horse fat. On the contrary, an investigator may want to participate in a study without fully realizing what tasks and obligations are involved in accurately and carefully conducting clinical research, and without showing a clear enthusiasm for the study protocol. The monitor should seriously doubt accepting this physician as an investigator, and possibly advise against it, as the investigator may well have perceived this study as a side activity, thereby endangering its success. • An investigational site should be fully aware of all the efforts that must be made to conduct a clinical study properly and successfully. • The investigator should be able to recruit a sufficient number of eligible subjects meeting the selection criteria. • The research staff – including the investigator – should be qualified by education, training, and experience. • The facilities and equipment should be adequate and validated where required. • And finally, the investigational site's infrastructure should be such that it can guarantee a timely conduct of the study according to good clinical practice rules.	
5. Should an investigational team conducting an investigator-initiated clinical study have a quality system with standard operational procedures (SOPs) describing the processes and products?	It is absolutely essential for all parties involved in any clinical research to have knowledge of their organization's SOPs. Each and every organization should describe its processes and procedures in a set of SOPs that apply to everybody within their organization. • These SOPs should include a listing of functional responsibilities and an overview of standard forms that apply to each process or procedure. • They should be available to each employee as a quality handbook. • SOPs should be part of the organization's quality system. • SOPs can be used for quality assurance and quality control purposes, as each employee should be familiar with the SOPs that apply to his/her tasks. • SOP knowledge may be tested on a regular basis in order to prove to third parties that the organization's employees are truly and fully qualified for the tasks that they have been assigned.	4.1
6. Where should we ideally organize an investigator meeting?	Whenever possible, training and instruction sessions of the sponsor's team and the investigational team should be organized in a place that is located as far away as possible from the participants’ day-to-day workplace. The essential training for both teams should consist of study product qualities and study product use, study procedures and timelines, the informed consent procedure, subject recruitment, CRF and source completion, and SAE reporting. • Training should be organized in an isolated and quiet location, away from (cellular) telephones.	5.7
7. Should an investigator read the investigator product brochure (IB)?	For any clinical study with an experimental medicinal product or radiopharmaceutical agent, the sponsor of the study is obliged to draft an investigator's brochure (IB) with all relevant and up-to-date non-clinical, preclinical, and clinical information about the product including safety and efficacy. Investigators have no passion to read the IB. Hence, they do not know the benefit/risk balance for the product in this stage of development. • The investigator should always invest time to become aware of the benefits and risks of the product. • He or she should be prepared for the following question, the patient can ask: ‘If your relative would have the same condition as I have, would you recommend them to participate in this study?’. Any investigator not knowing the risks will non-verbally communicate ‘doubt’. This will be noticed by the patient.	4.1.2.
8. What is the best way to teach a study protocol to the members of my investigational team?	Acting carefully can only be realized if the entire study protocol, including all tasks and obligations for the investigator and his/her team, has been cut up into bite-size chunks. • Cut the study protocol into bite-size chunks. • The study monitor and the principal investigator should make a practical translation of the study protocol that meets the factual requirements of the investigational site. Only if this is done, can a complete overview be made of the time that all the tasks will take, and the investigational team can conduct a detailed capacity calculation. Moreover, such a translation will also allow for a more accurate planning of timelines.	4.2.4.
9. What is the true value of an initiation visit?	Acting carefully means that the monitor should inform, instruct, and coach the investigational team during the initial phase of the study. In theory, during the site initiation visit, the investigational team is to be fully instructed about the study procedures. In the real practice, this is not possible. • As there has been no actual practical experience with the study yet, everything that is said during the initiation visit is theory and consequently people will listen with one ear. Moreover, there may be some overconfidence during the initiation visit. It all seems so familiar or simple. However, everybody should be aware that there might be pitfalls. In order to keep the investigational team's attention during the initiation visit, the monitor should actually revert to medieval practices. As this does not promote a good understanding or an effective working relationship, the initiation visit should be a short and clear meeting focusing on the main issues of the study. • As soon as two subjects have been included in the study, the monitor should repeat the initiation of the site thoroughly, thereby analyzing and discussing the mistakes that have been made directly with the investigator and the relevant staff. Thus, the initiation instruction is to be repeated during the first on-site monitoring visit. In addition, it may be advisable for the investigator to • Organize a dummy-run of the protocol-related procedures with his/her research staff prior to the start of the study.	8.2.20
10. When a physician invites a patient to participate in a clinical trial, the patient trusts that (s)he is asked with	This means that investigators should realize that they are 100% knowledgeable on the benefits and risks of the proposed trial and intervention. The investigator should always imagine the situation that he or she should ask a relative to participate in the study.	4.8.1., 4.8.5.
the best possible motive. What holds the responsibility of an investigator?		
11. Does the patient understand what the information on the study translates into real life?	The regulations are clear which information should be communicated to eligible individuals. Due to the vast amount of information that has to be included in the patient information form, one can speculate that the patient did NOT understand the information provided. However, taking several factors into account, including functional illiteracy, society should require that • Any text for patients to be assessed by a competent translation agency for layman instead of an expert in the Ethics Committee only.	4.8.6.
12. Will a patient admit that he or she does not (fully) understand the patient information?	A patient will always deny that (s)he did not understand the patient information. ‘When you admit that you do not understand the information provided, you communicate how stupid you are’. • That is why the informed consent procedure is a communication between two people and no more than that. Keep the information short and simple and take sufficient time to explain the situation, the background, the purpose, the tasks and obligations, and once done, ask the patient what (s)he has understood and if there are any questions left.	4.8.7., 4.8.9., 4.8.10.
13. Who is allowed to conduct an informed consent procedure in a clinical study according to current standards?	Carefulness with respect to the informed consent procedure means that both the investigator and a research nurse will conduct the procedure. In a hospital setting, it can be generally assumed that patients neither listen nor understand the detailed instructions given by the physician/investigator. A research nurse: Once patients leave the doctor's office, I always ask them what the doctor has told them. In most cases the patient replies: ‘I haven't the faintest idea’. Upon my question: ‘Did not the doctor ask you to participate in a clinical study and what kind of study it was?’, the patient mostly would answer: ‘Truly, I did not understand a word’. • The principal investigator and research nurse being aware of the selective perception of the patient, should both conduct the procedure. Patients tend to listen more attentively to research nurses, as nurses are closer to them. In addition, this approach also allows that both the investigator and the research nurses can decide more carefully on the eligibility of the patient for the study.	4.2.4.
14. How can the investigator ensure that the patient information sheet is simple enough for laymen?	Both the information and the informed consent form should be reviewed by a number of lay persons. • Take a member of your family who does not have anything to do with clinical research and ask them to tell you in their own words what they think is meant and requested. If they understand everything, it will be acceptable. This kind of validation is much more reliable than any review from knowledgeable and competent parties.	4.8.6., 4.8.10.
15. Is it difficult to obtain informed consent in a clinical study?	To obtain informed consent may seem a challenge; however, to maintain informed consent during the study is a much bigger challenge.	4.8.3.
	Once the patient has been enrolled in the study, (s)he may start to realize that much more has to be done for the trial than anticipated. During the study, the investigational team should invest time to reinforce the patient and to keep him/her updated about the ins and outs of the study.	
16. How can we motivate patients to keep them in the study?	In order to keep patients motivated to continue their participation in the study, the investigational team should build a proper relationship with the patient. Ask how (s)he copes with the study procedures, and how it fits in with their day-to-day lives (e.g. how are the children, how do you spend your holidays this year, etc.).	Common sense
17. Once enrolled, does a patient always need to come to the clinic during the study?	In a clinical study, it is custom that the patient visits the hospital or research center for any trial procedure. However, too many visits could become a burden to the patient. In that case, the investigational team should • Consider visiting the patient at home for trial procedures such as blood sampling and/or questionnaires. • It is better to invest some extra time, resources, and/or travel than losing patients due to excessive study burden.	4.3.2.
18. Why should the investigator report any deviation from the protocol, given the administrative burden?	In a randomized controlled clinical trial, the approved study protocol is considered sacred. Protocol deviations are only allowed when the situation for the patient becomes hazardous and should always be well-recorded by the investigator. Strict adherence to the study protocol is required, especially for the inclusion and exclusion criteria to avoid protocol violations. • An investigator should comprehend that any patient not complying to the study protocol will not be included in the per protocol analysis of the study. This could mean that a study cannot be properly analyzed when there are too many protocol violations.	4.5.1.
19. Most of the serious adverse events (SAEs) in a clinical study are related to the medical condition of the patient. Why should the investigator report these SAEs to the sponsor?	A SAE is any event that could lead to the death of the patient, a life-threatening situation, persistent or significant disability/incapacity, requires inpatient hospitalization or prolongation of existing hospitalization, or results in a congenital anomaly/birth defect. It is not relevant if there is an intended or unintended causal relationship to the intervention. Hence, the motto: ‘Do not think, just report the SAE to the sponsor’. • The biggest mistake an investigator can make in a clinical study is to assess a serious adverse event (SAE) as unrelated to the intervention and, therefore, not report the event to the sponsor of the study.	4.11.1

a ICH Harmonised Tripartite Guideline for Good Clinical Practice, Step 5, 1 May 1996 (CPMC 135/95) which became into operation as of January 1997.

## Summary

Early phase clinical interventional trials with new medications need a complex organization consisting of several general and dedicated SOPs and quality requirements in accordance with GCP. In this ever-changing landscape of (inter)national rules and regulations, it is wise that all those participating in clinical research keep their knowledge of these rules and regulations updated.

For this purpose, a local quality system should be built based on discussion with the organization on the mission, vision, tasks and responsibilities, services rendered, stakeholders involved, and so on. Each organization should make a quality manual describing all these aspects. This manual should always serve as a guide for the GCP inspection to assess the organization and its compliance to the rules and regulations in clinical research.

As a first step in the Netherlands, the Union of Universities started in 2007 with the organization of an intensive basic course on rules and regulations in clinical research for both new and experienced clinical investigators within academic, CRO, and pharma settings. This so-called ‘BROK’ course (Basic course for Rules, Regulations and Organization of Clinical Research for Clinical Investigators) is followed by an exam where the participants provide proof that they have sufficient knowledge to start an interventional clinical study in the Netherlands (4). Most Dutch Ethics Committees not only require a registered but also an accredited, principal investigator as a prerequisite to approve a clinical interventional study.

However, having such an accreditation is not enough, conducting clinical research in accordance with the rules and regulations requires the right scientific attitude and discipline to do the tasks: accurately, timely, documented, and in full honesty.
